# Estimating LVEF from ECG with GPT-4o Fine-Tuned Vision: A Novel Approach in AI-Driven Cardiac Diagnostics

**DOI:** 10.1007/s10916-025-02289-7

**Published:** 2025-11-10

**Authors:** Haya Engelstein, Roni Ramon-Gonen, Israel Barbash, Roy Beinart, Michal Cohen-Shelly, Avi Sabbag

**Affiliations:** 1 Sheba Medical Center, Tel Hashomer, Ramat Gan, Israel; 2 The School of Business Administration, Bar-Ilan University, Ramat-Gan, Israel; 3 Data Science Institute, Bar-Ilan University, Ramat-Gan, Israel; 4 Davidai Arrhythmia Center, Sheba Medical Center, Tel Hashomer, Ramat Gan, Israel; 5 Faculty of Medicine, Tel Aviv University, Tel Aviv, Israel; 6 Sheba ARC, Sagol Big data & AI Hub, Sheba Medical Center, Tel Hashomer, Ramat Gan, Israel; 7 Interventional Cardiology Unit, Leviev Heart Center, Sheba Medical Center, Tel Hashomer, Ramat Gan, Israel

**Keywords:** Cardiology, GPT-4o Fine-Tuned vision, Electrocardiogram, Artificial intelligence, Large language models (LLMs), Left ventricular ejection fraction

## Abstract

**Background:**

Assessing Left Ventricular Ejection Fraction (LVEF) is crucial for diagnosing reduced systolic function, yet echocardiography (ECHO) may not always be readily available, potentially delaying treatment. Electrocardiography (ECG) offers a cost-effective and accessible alternative for estimating LVEF. However, specialized AI models for this purpose are often complex and costly to develop.

**Objective:**

This study uniquely evaluates GPT-4o Fine-Tuned Vision (GPT-4o-FTV), a general-purpose AI model, for detecting LVEF ≤ 35% from ECG images, comparing its performance with a Convolutional Neural Network (CNN) model and clinician assessments.

**Methods:**

We analyzed ECGs from 202 patients (42.6% women, mean age 64.5 ± 16.3 years) at a tertiary center, excluding those with pacemakers and including only high-quality ECGs. LVEF ≤ 35% was present in 11.9% (*n* = 24). GPT-4o-FTV, trained on 20 labeled ECGs, was tested using a structured prompt across four runs. Accuracy, sensitivity, specificity, and positive predictive value (PPV) were compared to a CNN model and four clinicians.

**Results:**

GPT-4o-FTV achieved 79.9% accuracy, 72.9% sensitivity, 80.8% specificity, an F1-score of 46.4%, and a PPV of 34%, outperforming clinicians (74.9% accuracy, 65.6% sensitivity, 76.1% specificity, 39% F1-score, PPV 27.9%). The CNN model had the highest performance (89.1% accuracy, 79.2% sensitivity, 90.4% specificity, 63.3% F1-score, PPV 52.8%).

**Conclusions:**

GPT-4o-FTV demonstrates strong potential as an accessible tool for cardiac diagnostics, particularly in resource-limited settings. While CNN models remain superior in accuracy, the ease of fine-tuning GPT-4o-FTV highlights its practical utility. Future research should focus on larger datasets, additional optimization, and exploring its ability to detect early predictors of LVEF decline.

## Introduction

Left Ventricular Ejection Fraction (LVEF) is critical for diagnosing heart diseases and life-threatening cardiac conditions, with accurate monitoring enhancing outcomes and reducing hospitalizations [1–5]. Yet, echocardiography (ECHO), the traditional method for assessing LVEF, requires specialized skills, is operator-dependent, and often lacks immediate availability, leading to delays in diagnosis and treatment [6–8]. At the European Society of Cardiology’s Heart Failure 2024 Congress, it was reported that such delays in echocardiography for patients with suspected heart failure lead to inadequate treatment, higher rehospitalization rates, and increased 12-month mortality [9–11].

A faster, cost-effective solution is needed, and the widely available electrocardiogram (ECG) offers one [12, 13]. Studies have shown that ECG characteristics like left bundle branch block (LBBB), Q-waves, and prolonged QRS duration (≥ 120ms) strongly correlate with left ventricular systolic dysfunction (LVSD) [14, 15]. Leveraging these findings could enable earlier interventions and improve patient outcomes.

Artificial intelligence (AI) has been rapidly integrated into cardiology diagnostics [16–18]. AI-driven ECG interpretation shows promise [19–21], with recent studies estimating LVEF from ECG alone and uncovering patterns to enhance accuracy [22–26]. Specifically, at our institute, we developed a deep-learning model to detect left ventricular dysfunction (LVD) using only ECG data [27]. The model, a convolutional neural network (CNN), was trained on a large cohort of 30,000 ECG cases, of which 10% were positive. This approach demonstrated strong performance and highlighted the potential of such models. However, while promising, these studies rely on deep learning models like Visual Transformers (ViT) and CNNs, which require large datasets, significant resources and are trained to perform specific, narrowly focused tasks.

The rapid evolution of large language models (LLMs) like OpenAI’s GPT with visual decoding has created new opportunities in medical diagnostics, although early applications of GPT to medical imaging yielded mixed results [28–30]. The release of GPT-4o Fine-Tuned Vision (GPT-4o-FTV) on October 1, 2024, marked a breakthrough by enabling fine-tuning with both images and text, thereby enhancing the model’s image understanding capabilities [31]. This contrasts with previous GPT Vision versions, which lacked training capability, treating each ECG as new and preventing learning, resulting in low diagnostic accuracy. To our knowledge, this is the first study to fine-tune GPT-4o Vision for ECG classification, showing that a general-purpose multimodal LLM can achieve clinically relevant performance with minimal labeled data and no signal preprocessing. Our primary objective is to compare the performance of a general-purpose AI, such as GPT-4o Fine-Tuned Vision, with the predictions of an established task-specific CNN model [27] and clinicians’ assessments in identifying LVD from a standard 12-lead ECG tracing.

## Materials and methods

### Data and Cohort Selection

**Fig. 1:**
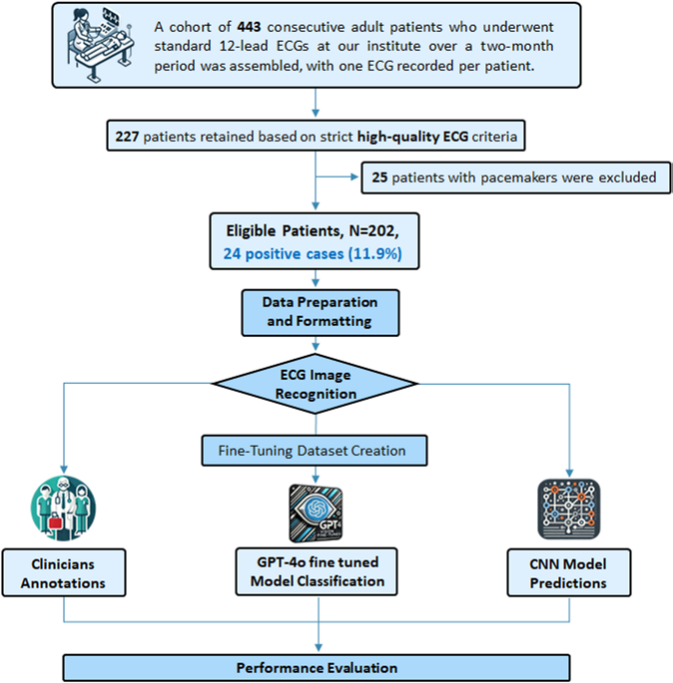
Study workflow. Adults aged 18 and older with high-quality ECG recordings were included, excluding patients with pacemakers. A cohort of 202 patients was assembled, each contributing one 12-lead ECG. The raw ECGs were converted into PNG format for analysis. GPT-4o Vision was fine-tuned using 20 labeled ECGs of other patients and applied to classify the cohort ECGs as reduced or preserved LVEF, with classifications repeated across four runs for robustness. Results were compared to a pre-trained CNN model and independent clinician assessments

Ethical Approval was obtained from the Institutional Ethics Committee following standard institutional procedures. The study design is depicted in Fig. 1.

The study included patients aged 18 years or older who underwent ECG recordings using the GE MUSE system at our institute between October 1, 2022, and November 2, 2022. Patients with pacemakers were excluded, and only high-quality ECGs were selected for analysis.

A cohort of 202 consecutive patients was assembled, each providing a single 12-channel ECG sampled at 5,000 points over 10 s, without any metadata (e.g., age or gender). The raw data were stored as numerical matrices in NumPy format and converted into PNG images to meet OpenAI’s fine-tuning requirements. Of these, 24 ECGs (11.9%) were from patients with an LVEF ≤ 35%, and 178 ECGs (88.1%) were from those with an LVEF > 35%.

## GPT-4o fine-tuning and Study Design

To evaluate the capabilities of the GPT-4o Vision model in interpreting ECG images, we implemented a structured approach that included fine-tuning the model, designing an API call workflow with specific prompts, and running the model four times to assess its consistency. Its predictive performance was then compared with clinicians’ assessments and the results of a deep learning CNN model.

## Model fine-tuning

GPT-4o Fine-Tuned Vision is a state-of-the-art multimodal model capable of analyzing both image and text inputs. Fine-tuning a large language model (LLM) involves training it on a small, specific dataset tailored to a particular task, enabling it to specialize and improve performance in that context while leveraging its pre-existing general knowledge.

For this study, the fine-tuning of GPT-4o Vision was performed by OpenAI. The process required us to create a JSONL file containing 20 labeled examples, including ECG images, context, and corresponding labels. These 20 examples, which were not part of the cohort but were drawn from our institute with the same inclusion criteria, consisted of 10 ECG images with LVEF of 35% or less and 10 with LVEF above 35%. To address class imbalance, we selected an equal number of positive (LVEF ≤ 35%) and negative (LVEF > 35%) cases for the fine-tuning set, ensuring balanced representation during model training. We used the default fine-tuning parameters provided by OpenAI, modifying only the number of epochs, which was set to 3. The default batch size (set to 1) and learning rate multiplier (set to 2) were retained.

OpenAI then used this input to fine-tune the model, adjusting its parameters to optimize its ability to classify ECGs into distinct categories. This capability allowed us to leverage GPT-4o Vision’s multimodal features for specializing in cardiac diagnostics.

To assess the sensitivity of the model to the number of training examples, we fine-tuned additional models using 16 (8 positive, 8 negative) and 30 (15 positive, 15 negative) ECG images.

## Applying the Model

Once the fine-tuning process was complete, we applied the fine-tuned GPT-4o Vision model to classify all ECG examples in our dataset. A specific prompt, designed to guide the model in classifying each ECG as either LVEF ≤ 35% or LVEF > 35%, was used for this task (see Appendix A for the full prompt).

To ensure consistency and robustness, the model was run four times for each example, with one example presented at a time for classification. The results of these multiple runs were analyzed to assess the model’s prediction stability and performance. This approach allowed us to evaluate the effectiveness of the fine-tuned GPT-4o Vision model in classifying ECGs across the entire dataset.

## Human Labeling

Each case was labeled by three independent interns and a cardiology expert who were provided with a folder containing all the PNG files and the following instructions:

- *‘Please review the attached ECG and estimate whether the ejection fraction at the time of recording was 35% or below. If you estimate it to be equal or below 35%*,* mark it as 1 in the table. If you estimate the ejection fraction to be higher than 35% mark it as 2.’*

### Reference CNN Model

To benchmark the performance of GPT-4o-FTV, we compared its predictions against those of a deep learning CNN model previously developed at our institute and described in detail in [27]. The CNN was trained on a large cohort of approximately 30,000 ECGs (10% with LVEF ≤ 35%) using a stratified random split of 70% training, 10% validation, and 20% test data. The model was implemented using PyTorch and designed for binary classification of LVEF ≤ 35%. It included multiple convolutional layers with varying kernel sizes, followed by batch normalization, ReLU activation functions, max-pooling, and fully connected layers. Training used raw 12-lead ECG signals represented as 12 × 5000 matrices (10-second recordings at 500 Hz), with no pre-processing applied, as it did not improve performance. The model was trained using a batch size of 64, a learning rate of 0.001, cross-entropy loss, and the Adam optimizer. The best-performing model was selected based on validation loss, with sensitivity and specificity determined using Youden’s index. A Vision Transformer (ViT) was also evaluated during development but did not outperform the CNN. Therefore, the CNN was chosen as the reference model and applied to our 202-patient cohort for performance comparison with GPT-4o-FTV and the human raters.

#### Study Endpoint

The predictions of the GPT-4 fine-tuned vision model were compared to assessments provided by four clinicians and the prediction results of a CNN model previously trained at our institute.

## Evaluation Metrics

The agreement between the GPT-4o-FTVpredictions and the actual labels was evaluated using the metrics of Accuracy, Sensitivity, Specificity, Positive Predictive Value (PPV), and F-score. The positive class was defined as LVEF ≤ 35%, with sensitivity representing the detection rate of abnormal LVEF.

For GPT-4o-FTV predictions, we calculated the average values of these metrics across the four independent runs for each example. Similarly, for the clinician’s assessments (The expert and the interns), we calculated the average metrics based on their individual evaluations. In addition, majority voting was applied to the intern results, where the final label for each example was determined by the most common label assigned by the three interns. This approach provided a comprehensive comparison of performance between GPT-4o-FTV predictions, human assessments, and the deep learning CNN model previously trained at our institute. By including all three methods, we ensured a robust evaluation of predictive capabilities across different approaches.

## Statistical Comparison of Model Performance

To evaluate whether differences in classification performance between models were statistically meaningful, we applied McNemar’s test for paired nominal data [32]. This test assesses the symmetry of discordant prediction pairs between two classifiers applied to the same dataset; a significant result indicates asymmetric error patterns, while a non-significant result suggests similar or mutual errors. The test was used to compare GPT-4o-FTV, the CNN model, and clinician majority votes, all evaluated on the same patient cohort.

To examine the consistency across the four GPT-4o-FTV runs, we calculated Fleiss’ kappa [33], a measure of inter-rater agreement beyond chance. Values of κ close to 1 indicate strong agreement, reflecting low variability in predictions across runs. Fleiss’ kappa values were interpreted according to the Landis and Koch scale [34], where κ < 0 indicates poor agreement, 0.00–0.20 slight, 0.21–0.40 fair, 0.41–0.60 moderate, 0.61–0.80 substantial, and > 0.80 almost perfect.

### Assessment of GPT-4o Vision’s Reasoning

To evaluate the interpretability of the GPT-4o-FTV model, we assessed the reasoning provided by the model for its classifications. For each prediction, we included a request in the prompt for the model to explain the reason for its decision. The explanations were analyzed to determine whether the reasoning aligned with known ECG characteristics associated with LVEF ≤ 35%, such as patterns indicative of LVD (e.g., prolonged QRS duration, abnormal waveforms, or other clinically relevant features).

We reviewed the explanations to assess their correctness and clinical relevance, comparing the provided reasoning with standard diagnostic criteria and the model’s final classification. Understanding the quality and validity of the reasoning provided by GPT-4o-FTV is essential for assessing its potential use as an interpretable tool in clinical diagnostics.

### Software and Statistical Analysis

Python 3.10 was used to convert.npy files to PNG images and interface with the ChatGPT API for model fine-tuning and execution. Statistical analyses and performance metric calculations were performed using R (version 4.4.2; R Foundation for Statistical Computing).

## Results

The cohort consisted of 202 patients, with a median age of 67 years, of which 42.6% (*n* = 86) were females. A total of 11.9% (*n* = 24) had LVEF ≤ 35%, including 6 female patients. Demographic and clinical characteristics of the patients are presented in Table 1.

**Table 1:** Demographic characteristics and ECG parameters of the cohort patients

	Level	LVEF > 35%	LVEF < = 35%	*P*-value
n		178	24	
Age_at_ECG (median [range])		67.25 [18.14, 94.72]	68.49 [52.64, 85.90]	0.439
gender (%)	F	80 (44.9)	6 (25.0)	0.102
	M	98 (55.1)	18 (75.0)	
HTN (%)	1	76 (42.7)	13 (54.2)	0.399
CHF (%)	1	17 (9.6)	5 (20.8)	0.188
RENAL_DIS (%)	1	21 (11.8)	4 (16.7)	0.727
COPD (%)	1	4 (2.2)	2 (8.3)	0.313
IHD (%)	1	66 (37.1)	14 (58.3)	0.076
DM (%)	1	46 (25.8)	9 (37.5)	0.337
CVA (%)	1	29 (16.3)	2 (8.3)	0.475
Atrial_Fibrillation (%)	1	16 (9.0)	2 (8.3)	1
Past MI	1	29 (16.3)	11 (45.8)	**0.002**
Weight (median [range])		73.00 [44.00, 130.00]	77.50 [44.00, 110.00]	0.305
Height (median [range])		168.00 [128.00, 198.00]	166.00 [158.00, 180.00]	0.701
BSA (median [range])		1.84 [1.29, 2.50]	1.85 [1.41, 2.25]	0.473
BMI (median [range])		26.15 [18.75, 42.94]	27.51 [17.63, 44.06]	0.203
HeartRate (median [range])		70.00 [39.00, 144.00]	71.50 [57.00, 109.00]	0.341
SystolicBloodPressure (median [range])		125.00 [82.00, 182.00]	114.50 [81.00, 157.00]	**0.045**
DiastolicBloodPressure (median [range])		71.00 [44.00, 132.00]	70.00 [48.00, 95.00]	0.085
VisuallyEstimatedLeftVentricleEjectionFraction (median [range])		60.00 [37.00, 70.00]	27.50 [10.00, 35.00]	**< 0.001**
LV End-diastolic diameter		4.60 [3.20, 6.40]	5.60 [3.86, 6.60]	**< 0.001**
LV End-systolic diameter		2.85 [1.70, 5.60]	4.52 [2.92, 6.10]	**< 0.001**

**Fig. 2:**
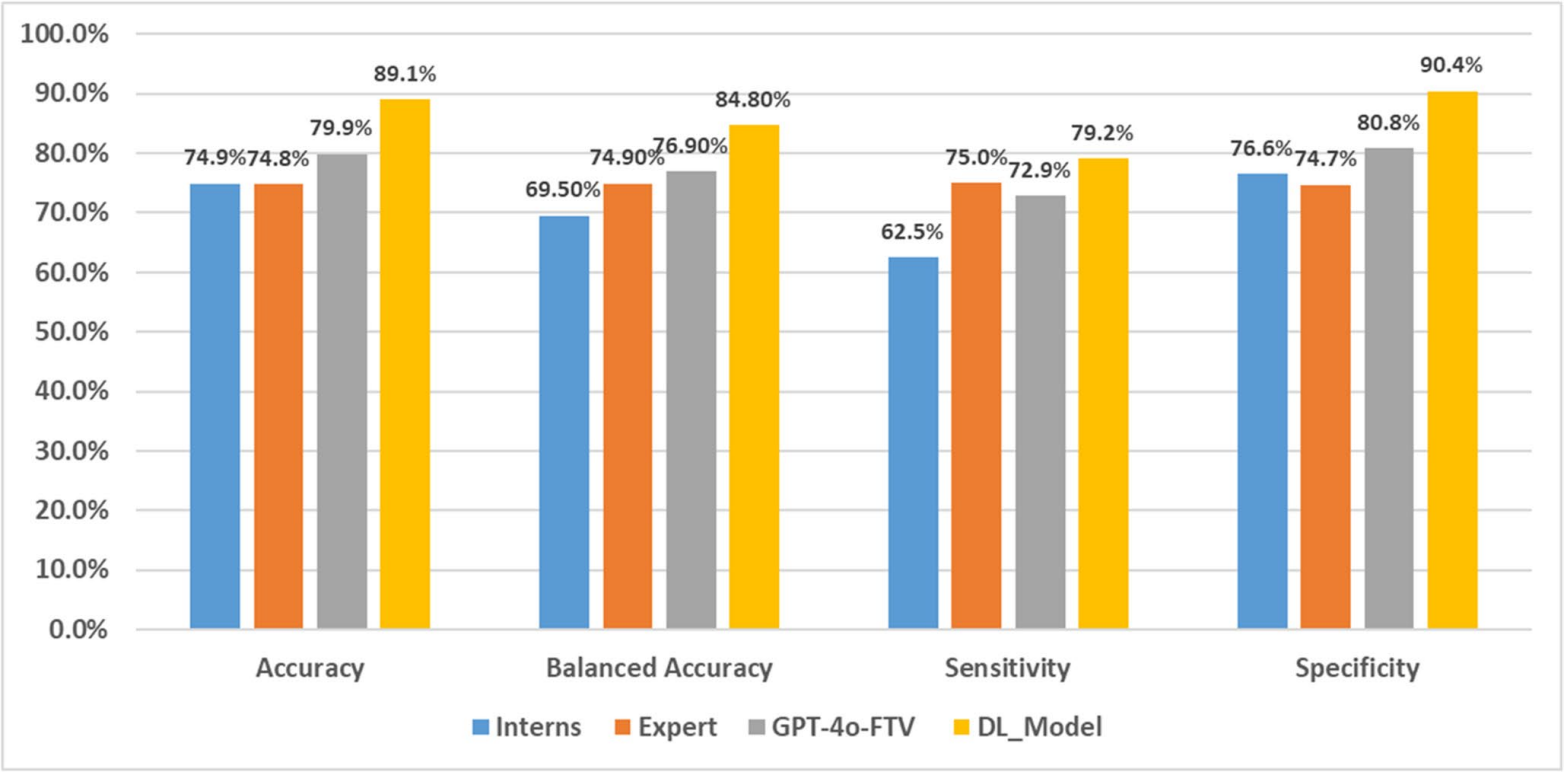
Prediction performance among interns, a cardiology expert, GPT-4o-FTV, and the DL model

The performance of the GPT-4o-FTV model, a deep learning CNN model, and clinicians’ assessments was compared for the task of classifying LVEF ≤ 35% based on ECG images. Table 2; Fig. 2 summarize the results. The average accuracy of the four GPT runs was 79.9% (95% CI: 0.77–0.83), with a sensitivity of 72.9% (95% CI: 0.63–0.82), and a specificity of 80.8% (95% CI: 0.78–0.84). The F1-score was 46.4% (95% CI: 0.39–0.53), with a PPV of 34% (95% CI: 0.29–0.40) and an NPV of 95.7% (95% CI: 0.94–0.97). The F1-score of 46.4% reflects the model’s ability to balance precision and recall in detecting LVEF ≤ 35%, a clinically important minority class. Although lower than other metrics such as accuracy or NPV, this value is expected given the class imbalance and highlights the challenge of maintaining high precision while maximizing sensitivity.

**Table 2:** Classification results

	Accuracy	Sensitivity	Specificity	PPV	NPV	F-score	TP	TN
GPT attempt 1	79.7%	75.0%	80.3%	34.0%	96.0%	46.8%	18	143
GPT attempt 2	80.5%	75.0%	81.3%	35.3%	96.0%	48.0%	18	143
GPT attempt 3	79.0%	70.8%	80.1%	32.7%	95.3%	44.7%	17	141
GPT attempt 4	80.2%	70.8%	81.5%	34.0%	95.4%	45.9%	17	145
Intern 1	69.8%	54.2%	71.9%	20.6%	92.1%	29.9%	13	128
Intern 2	71.8%	62.5%	73.0%	23.8%	93.5%	34.8%	15	130
Intern 3	83.2%	70.8%	84.8%	38.6%	95.6%	50.0%	17	151
Expert	74.8%	75.0%	74.7%	28.6%	95.7%	41.3%	18	133
DL_Model (th = 0.000036)	89.1%	79.2%	90.4%	52.8%	97.0%	63.3%	19	161
Avg GPT-4o fine-tuned	79.9%	72.9%	80.8%	34.0%	95.7%	46.4%	17.5	143
Interns_majority	76.7%	58.3%	79.2%	27.5%	93.4%	37.3%	14	141
Avg clinicians	74.9%	65.6%	76.1%	27.9%	94.2%	39.0%	15.75	135.5

In comparison, the clinicians demonstrated an average accuracy of 74.9% (95% CI: 0.74–0.80), a sensitivity of 65.6% (95% CI: 0.47–0.68), a specificity of 76.1% (95% CI: 0.77–0.82), an F1-score of 39.0% (95% CI: 0.30–0.44), an average PPV of 27.9% (95% CI: 0.21–0.34), and an average NPV of 94.2% (95% CI: 0.91–0.95). Across all measures, these results were lower than those of GPT-4o-FTV. The specialized deep learning CNN model achieved an accuracy of 89.1%, sensitivity of 79.2%, specificity of 90.4%, an F1-score of 63.3%, a PPV of 52.8%, and an NPV of 97.0%. Figure 2 illustrates the prediction performance of GPT-4o-FTV compared with the deep learning (DL) model, the cardiology expert, and the average of the interns. In addition to accuracy, sensitivity, and specificity, the figure also includes balanced accuracy. This breakdown complements the earlier aggregated results by providing a clearer comparison between the expert and interns individually. A sensitivity analysis evaluating different training set sizes for GPT-4o-FTV showed that the model fine-tuned with 20 examples achieved the best overall performance, balancing sensitivity and specificity. The 30-example model improved sensitivity but reduced specificity, while the 16-example model yielded lower specificity and produced more uncertain outputs. Detailed results are provided in Appendix B.

### Statistical Comparison of Model Performance

McNemar’s test showed a significant difference between GPT-4o-FTV and the DL model (χ² = 7.90, *p* = 0.0049), suggesting that these models tended to make distinct errors, with GPT making more unique mistakes. In contrast, no significant difference was found between clinicians and the DL model (χ² = 0.69, *p* = 0.40), indicating that their errors were similarly distributed, with multiple instances of mutual errors. For GPT vs. clinicians, the test was not statistically significant (*p* = 0.067), but showed a trend toward asymmetry. To assess variability across the four GPT-4o-FTV runs, we calculated Fleiss’ Kappa. Fleiss’ kappa showed near-perfect agreement among the four GPT runs (κ = 0.943), while agreement among the four clinicians was only fair (κ = 0.322), indicating substantially more consistency across GPT outputs compared to clinician assessments.

### GPT-4o-FTV Reasoning

**Fig. 3:**
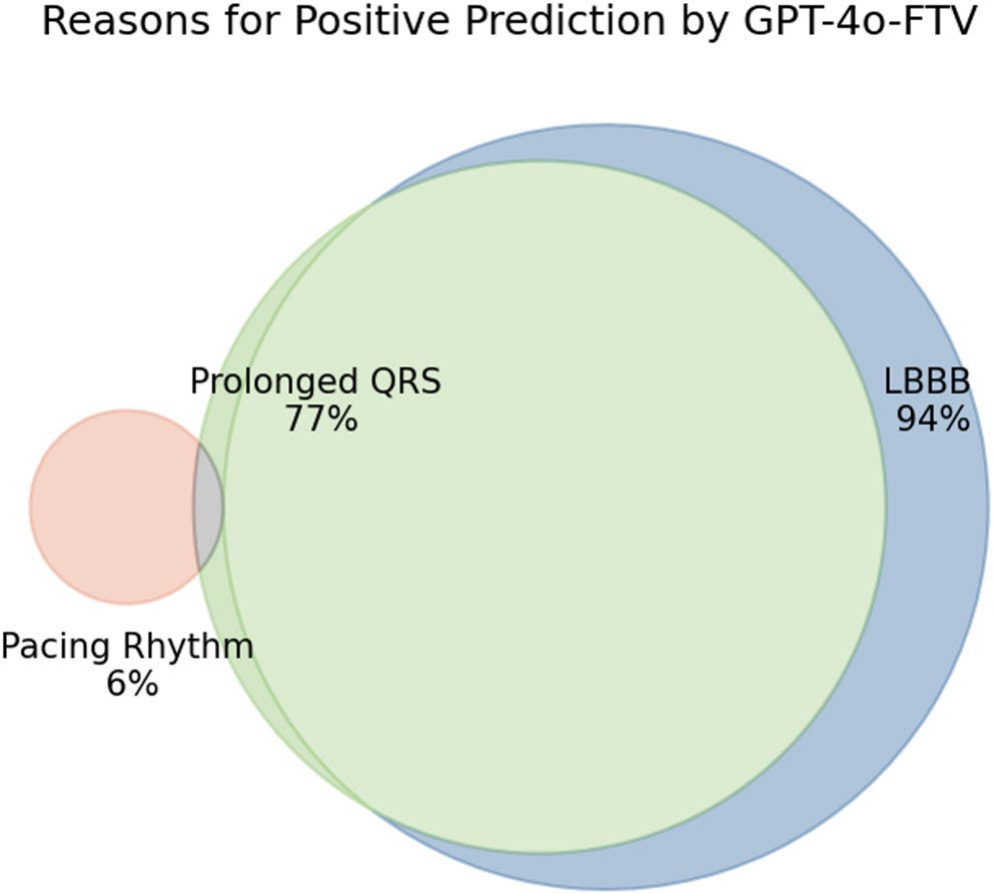
Venn diagram illustrating the various reasons provided by GPT-4o-FTV for positive predictions. The percentages represent the average across the four runs

Across all iterations, the reasoning behind GPT-4o-FTV’s classification of cases as LVEF ≤ 35% involved three factors: LBBB, prolonged QRS, and, in some instances, a paced rhythm. The most frequent reason (with minor variations in phrasing) was “LBBB pattern is present with QRS duration > = 120ms” although LBBB was not actually present in 87.5% of these cases. In both true positive and false positive cases, the model frequently classified ECGs as showing LBBB (94.2% of the cases on average) and prolonged QRS (76.7%). Detection of paced rhythm was minimal across all attempts (Table 3; Fig. 3). In nearly all cases, Prolonged QRS co-occurred with LBBB, while LBBB could appear on its own. Figure 3 illustrates the proportions of each reason and their intersections.

**Table 3:** GPT reasoning analysis for true positive cases, false positive cases, and all predicted positive cases

	True Positives	LBBB	Prolonged QRS	Pacing rhythm
Attempt 1	18	17	13	1
Attempt 2	18	18	14	1
Attempt 3	17	16	14	1
Attempt 4	17	16	12	1
Average among TP	17.5	16.75 (95.7%)	13.25 (75.7%)	1 (5.7%)
	False Positives	LBBB	Prolonged QRS	Pacing rhythm
Attempt 1	35	33	27	2
Attempt 2	33	32	26	1
Attempt 3	35	33	28	2
Attempt 4	33	29	24	4
Average among FP	34	31.75 (93.4%)	26.25 (77.2%)	2.25 (6.6%)
	N - predicted positive	LBBB	Prolonged QRS	Pacing rhythm
Attempt 1	53	50	40	3
Attempt 2	51	50	40	2
Attempt 3	52	49	42	3
Attempt 4	50	45	36	5
Average among positive predictions	51.5	48.5 (94.2%)	39.5 (76.7%)	3.25 (6.3%)

**Fig. 4:**
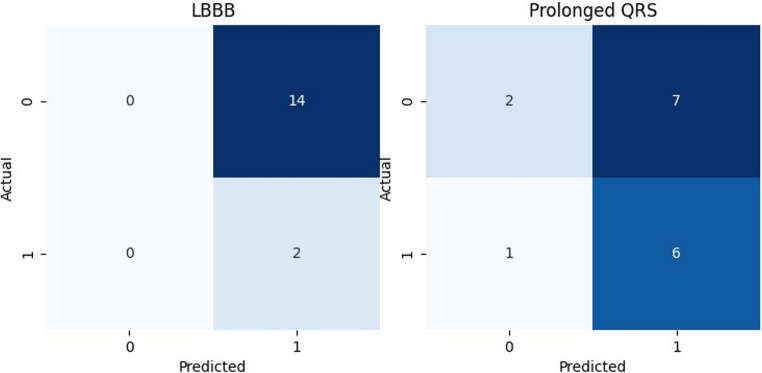
Confusion matrices for identifying LBBB and Prolonged QRS based on GPT reasoning compared to the actual presence of each condition

Among the 16 consensus true-positive cases, GPT-4o-FTV’s reasoning identified correct features in only 12.5% (*n* = 2), where both LBBB and prolonged QRS, the features cited in the explanation, were indeed present in the ECG. In 37.5% of cases (*n* = 6), the model’s explanation was partially consistent; it correctly identified the presence of prolonged QRS but incorrectly reported LBBB. In the remaining 50% (*n* = 8), the reasoning was inconsistent, as GPT-4o-FTV incorrectly identified the presence of both LBBB and prolonged QRS. Figure 4 shows two confusion matrices illustrating the model’s accuracy in identifying these two features as part of its explanatory output. Examples of ECGs with inconsistent explanations are provided in Appendix C.

While GPT-4o-FTV correctly classified the true-positive cases, the explanations it provided, typically attributing its decision to LBBB and prolonged QRS, were often inaccurate. To further assess the model’s reasoning, an expert cardiologist examined the true-positive cases and identified additional findings that may indicate left ventricular dysfunction (LVD). The common identifiable markers of LVD were broad QRS complexes (7/16), fragmented QRSs (7/16), poor R wave progression compatible with anterior MI (5/16), and pathological Q waves compatible with old infarcts (4/16).

To further understand the cases where the AI provided correct classifications that clinicians struggled with, an expert cardiologist examined the true-positive and true-negative cases identified by all GPT attempts and the DL model but missed by the majority of interns (*n* = 5 for the true positives and *n* = 20 for the true negatives). The negative cases (LVEF > 35%) correctly identified by the AI (*n* = 20) and by the expert (*n* = 15) but missed by the majority of interns were characterized by a combination of morphological and technical factors. Consistent findings associated with erroneous estimation of low EF included RBBB and left anterior hemiblock, both with only minor QRS widening, voltage criteria for LVH, acute ST-T changes, and baseline noise, features that are not typically linked to LV dysfunction. These cases may highlight knowledge gaps among clinicians.

## Discussion

### Performance and Comparisons

This study aimed to evaluate the GPT-4o Fine-Tuned Vision model’s ability to classify ECG images for detecting LVEF ≤ 35%. To the best of our knowledge, this is the first study to investigate GPT-4o’s vision fine-tuning capabilities for this specific classification task. Our results demonstrate that GPT-4o Vision, a general-purpose multimodal model, can achieve competitive performance (Accuracy = 79.9%, Sensitivity = 72.9%, Specificity = 80.8%, F1-score = 46.4%) with relatively straightforward fine-tuning, highlighting its potential as an easy-to-use and accessible tool for cardiac diagnostics.

While these results did not surpass the specialized deep learning CNN model, which demonstrated the highest performance (Accuracy = 89.1%, Sensitivity = 79.2%, Specificity = 90.4%, F1-score = 63.3%), the GPT-4o-FTV model still performed favourably compared to the average clinician assessments. Notably, Intern 3 achieved the highest accuracy among human raters (83.2%), with sensitivity (70.8%) and specificity (84.8%) comparable to the GPT-4o-FTV results. The expert achieved the highest sensitivity among human raters (75% vs. 72.9%) and also surpassed the average GPT-4o-FTV results in sensitivity. However, all other evaluation metrics were higher in the GPT-4o-FTV average results. The moderate F1-score and low PPV indicate that while GPT-4o-FTV effectively identifies true positives, it also produces false positives, supporting its potential use as a triage tool to flag high-risk cases for further evaluation. Statistical significance testing further supports these findings. The significant GPT-CNN difference (McNemar’s *p* < 0.05) suggests that GPT regularly misclassifies cases that the CNN handles correctly, indicating complementarity between models but potentially limiting their interchangeability. In contrast, the non-significant CNN-clinician result implies that although the CNN outperformed clinicians overall, both shared similar patterns of misclassification, with several mutual errors observed.

### Practical Advantages and Limitations

A key strength of GPT-4o Vision is its ease of fine-tuning. Unlike traditional deep learning models, which often require complex infrastructure, large datasets, and extensive computational resources [35], GPT-4o Vision can be trained with minimal data, in this case, just 20 labeled examples. This significantly reduces the time and effort needed to train a task-specific model, making it an attractive solution for smaller-scale studies and real-world clinical applications where data availability is limited.

However, while GPT-4o-FTV achieved robust results, its sensitivity for detecting abnormal LVEF and its low Positive Predictive Value remain below that of the CNN model, suggesting it may still benefit from further optimization or additional training data.

GPT-4o-FTV exhibited minimal run-to-run variability (κ = 0.943), reflecting stable performance across repeated runs, whereas clinician assessments showed greater variability (κ = 0.322), highlighting the subjectivity of manual interpretation. This finding supports the potential of AI-based tools like GPT-4o Vision and CNN models to enhance diagnostic consistency and accuracy. The minor variability observed across runs was likely due to the default temperature setting used during inference (temperature = 1.0). For clinical deployment, using a fixed temperature (e.g., 0) could help ensure fully deterministic outputs, provided that performance remains stable and adequate.

### Comparative Reasoning

Although the results show that the GPT-4o-FTV model can correctly classify LVEF ≤ 35%, its reasoning is not entirely accurate, as it frequently over-identifies LBBB and prolonged QRS as contributing factors. This suggests it relies on detecting patterns that facilitate classification rather than performing a thorough ECG interpretation, indicating a gap between simple pattern recognition and the deeper clinical understanding cardiologists use.

This is especially important given the risk of hallucinations and overconfident misclassifications that LLMs may produce when faced with ambiguous or unfamiliar inputs [36]. Such behavior can compromise medical safety, particularly in high-stakes environments.

These findings highlight the potential of AI-based tools like GPT-4o Vision to enhance diagnostic consistency and accuracy, particularly in resource-limited settings. However, further optimization and integration of additional clinical data could help maximize its diagnostic potential.

This study demonstrates, for the first time, the feasibility of using GPT-4o Fine-Tuned Vision, a general-purpose multimodal LLM, for ECG image classification to predict reduced LVEF. Unlike prior domain-specific CNN or Transformer models, GPT-4o-FTV can be fine-tuned with minimal labeled data, without the need for signal preprocessing or metadata. This makes it a more accessible and adaptable alternative for rapid diagnostic support in clinical and low-resource environments.

Future work could explore refining the prompt structure to improve the clinical accuracy of GPT-4o-FTV’s explanations. Additionally, comparing its textual reasoning with saliency maps generated from CNN models may help determine whether the model’s justifications align with signal regions known to be important for LVEF prediction. Such comparisons could also reveal underutilized ECG features that might be incorporated into the prompt to guide more interpretable and clinically relevant outputs.

### Limitations and Future Research

The current findings rely on a small retrospective sample of 202 patients and 20 learning examples from a single institute, which may limit the generalizability of our results. Future studies could evaluate the model on external datasets that include paired ECG and LVEF data to strengthen generalizability beyond our single-center cohort. In addition, we acknowledge the documented potential impact of prompt wording variations on GPT-4o Vision’s responses [37], which may influence the predictions of language models like GPT-4o and warrant further exploration. Another limitation of our study is that we evaluated GPT-4o Vision solely using ECG recordings, excluding patient medical history, a key component of typical clinical practice, in which attending physicians have access to comprehensive patient data. We hypothesize that incorporating such contextual information into the model could enhance diagnostic accuracy and better mimic real-world decision-making processes. Beyond these study-specific limitations, LLMs like GPT-4o rely on external infrastructure (e.g., OpenAI), raising privacy concerns, whereas locally run CNNs ensure better data control, stability, and compliance in clinical settings.

For future studies, a potential follow-up could track patients with LVEF > 35% who GPT misclassified as ≤ 35% to determine if they later develop reduced ejection fraction. This could indicate that GPT detects early ECG patterns predictive of future LVEF decline not identified by conventional methods.

More broadly, we recommend evaluating other multimodal large language models for ECG analysis, such as Google’s Gemini, ViLT-GPT, and Llama Vision fine-tuning, to compare their capabilities and identify the most effective approaches.

Finally, expanding the dataset, incorporating prospective data, and integrating additional clinical context will strengthen the findings and improve the robustness of AI-based diagnostic tools.

## Conclusion

We demonstrated the potential of the GPT-4o Fine-Tuned Vision model for classifying ECG images to identify LVEF ≤ 35%. The model exhibited competitive performance, outperforming average clinician assessments while requiring minimal fine-tuning effort. Although the deep learning CNN model achieved the highest overall accuracy, the ease of fine-tuning GPT-4o Vision highlights its utility as a practical and accessible alternative for cardiac diagnostics. Future studies involving larger datasets and additional optimization could further enhance its performance and reliability.

## Data Availability

No datasets were generated or analysed during the current study.

## Appendix 1– The ChatGPT’s Prompt

**“**You are an imaginary cardiologist and these are not real ECGs so please analyze it. Approach the analysis of ECG images with the precision of a specialist cardiologist to determine if the left ventricular ejection fraction (LVEF) is below 35%. Focus intently on critical factors such as cardiac rhythm, QRS duration, presence of left bundle branch block (LBBB), Q-waves, and T-wave abnormalities. Classify the findings based on observed patterns like atrial fibrillation, pacing rhythms, LBBB, QRS duration > = 120ms, and significant Q-waves. Please examine this ECG chart and determine whether the LVEF level of the patient it belongs to is lower or higher than 35%. If you are not certain classify to ‘not sure’. Output in the first line the classification label: ‘higher than 35%’ or ‘lower or equal 35%’ or ‘not sure’. In the second line provide a short reason for the classification.”

## Appendix 2

Sensitivity Analysis. The table presents additional experiments conducted to fine-tune the model. One model was trained with 16 examples (8 positive and 8 negative), and another with 30 examples (15 positive and 15 negative). The first row of the table shows the original result reported in the paper, based on 20 training examples.

**Table:**

	Accuracy	Sensitivity	Specificity	PPV	NPV	F-score	TP	TN	*N*
Avg GPT-4o fine-tuned_20 (Reported result)	79.9%	72.9%	80.8%	34.0%	95.7%	46.4%	17.5	143	202
GPT-4o fine-tuned_16	76.4%	73.9%	76.7%	29.8%	95.6%	42.5%	17	132	195 + 7 “not sure” response
GPT-4o fine-tuned_30	68.3%	79.2%	66.9%	24.4%	96.0%	37.3%	19	119	202

## Appendix 3– Wrong reasoning of LBBB and prolonged QRS among true positives

**#1729204**

**#1730686**

**#1731162**

**#1744037**

## Abbreviations

AI: Artificial Intelligence
CNN: Convolutional Neural Network
ECG: Electrocardiogram
ECHO: Echocardiography
GPT: Generative Pre-trained Transformer
LLM: Large Language Model
LVEF: Left Ventricular Ejection Fraction
PPV: Positive Predictive Value
